# Error mitigation in brainbox quantum autoencoders

**DOI:** 10.1038/s41598-024-84171-z

**Published:** 2025-01-17

**Authors:** Joséphine Pazem, Mohammad H. Ansari

**Affiliations:** 1 Peter Grünberg Institute (PGI-2), Forschungszentrum Jülich, 52428 Jülich, Germany; 2 Institut für Theoretische Physik, Universität Innsbruck, Technikerstraße 25, A-6020 Innsbruck, Austria; 3 Institute for Quantum Information, RWTH Aachen University, D-52056 Aachen, Germany

**Keywords:** Qubits, Information theory and computation

## Abstract

Quantum hardware faces noise challenges that disrupt multiqubit entangled states. Quantum autoencoder circuits with a single qubit bottleneck have demonstrated the capability to correct errors in noisy entangled states. By introducing slightly more complex structures in the bottleneck, referred to as brainboxes, the denoising process can occure more quickly and efficiently in the presence of stronger noise channels. Selecting the most suitable brainbox for the bottleneck involves a trade-off between the intensity of noise on the hardware and training complexity. Finally, by analysing the Rényi entropy flow throughout the networks, we demonstrate that the localization of entanglement plays a central role in denoising through learning.

## Introduction

Quantum computing holds the promise of surpassing classical computing paradigms^1–3^. However, the performance of Noisy Intermediate-Scale Quantum (NISQ) processors^4^ is significantly hindered by various infidelities and noise-induced challenges^5–10^. The preparation of multi-qubit entangled states on these processors is particularly vulnerable to errors, such as bit-flips^11^. Effective denoising requires meticulous characterization of noise sources and the development of protocols that mitigate these disturbances without altering the quantum state’s essential properties. Advancements in state denoising mark one of the critical milestones toward realizing scalable and fault-tolerant quantum computing. There are several deterministic protocoles to address denoising: Quantum error correction (QEC) schemes aim to detect and rectify such errors in logical qubits^12,13^. Entanglement purification protocols also enhances fidelity by filtering out erroneous states from a subsystem^14^. Progress has also been made through thermal cluster states, which exploit phase transitions from infinite to finite entanglement lengths^15^. Furthermore, topological quantum error-correcting codes offer robust solutions by encoding information in protected, non-local degrees of freedom^16^.

Machine learning (ML) offers a powerful tool for automating denosing process by enabling adaptive and data-driven approaches to extract required information from noisy data^17^. Classical ML identifies statistical patterns in real number data^18,19^, however it unables to deal with complex distributions of entangled states in quantum theory^20^. Quantum machine learning (QML) offers potential improvements in processing these distributions^21–24^, though true quantum speedups require ideal conditions not yet realized in current devices^3,10,25–27^.

Quantum autoencoders (QAEs) have demonstrated significant potential for denoising and data compression on NISQ devices^28–30^. By compressing data into a latent space, QAEs effectively learn to reconstruct ideal quantum states from noisy inputs-a task challenging for deterministic methods due to their sensitivity to inherent noise. Unlike algorithmic state preparation, which deterministically produces desired state by transforming a pure initial state (e.g., $|0\rangle ^{\otimes n}$), QAEs employs unsupervised learning to automate noise correction^30,31^. Their success in mitigating noise in entangled states makes them useful in metrology, error correction, and state preparation^31–35^.

The application of QAEs extends beyond state preparation to other areas, such as Quantum Secret Sharing (QSS)^36^. In QSS, a secret is distributed among participants, where individual shares reveal nothing alone, but together they disclose the full secret. For example, a GHZ triplet is shared among Alice, Bob, and Charlie, who each measure in a chosen basis to use as shared secret. The GHZ triplet affected by noise has lower success probability for secret recovery, which significantly declines by noise strength^37^. QAEs can counteract this decline by denoising the GHZ triplet before distribution, effectively reducing error rates and enhancing QSS security.

**Fig. 1:**
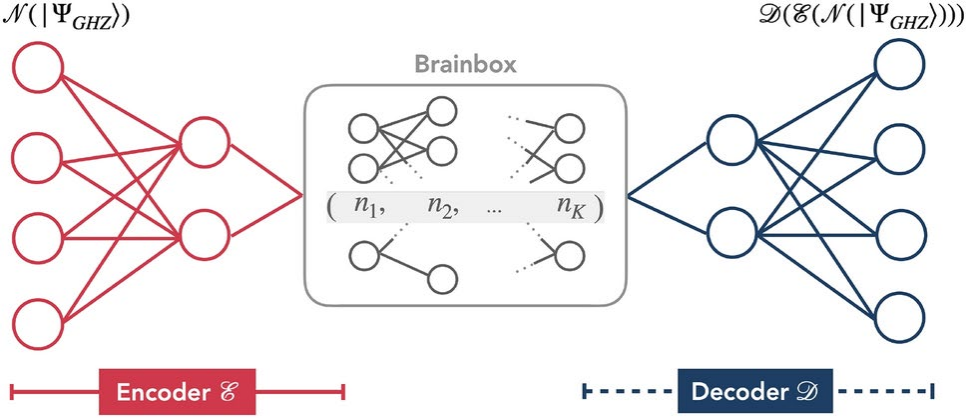
Architecture of a typical brainbox quantum autoencoder with symmetric 4-qubit input/output layers and the brainbox of K layers in the middle. The left red subnet encodes the state of input layer on BB by compressing it and the right blue subnet decodes the compressed state on the output layer. BB is represented by the set of qubit numbers in a row from left to right, i.e., $(n_1, \cdots , n_K)$. For example, (1, 1, 1) means -QAE, (2) is -QAE, and (1, 2) is -QAE.

In this paper we extend the Quantum Autoencoder (QAE) framework by introducing a “Brainbox Bottleneck (BB),” a sublattice of qubits that replaces the conventional single-qubit bottleneck to define the latent space. These brainbox circuits, which can vary in layout and consist of one or multiple layers (see Fig. 1), provide enhanced flexibility and robustness. Notably, certain BB-QAEs demonstrate significantly higher resilience to strong noise compared to conventional single-node QAEs^30^. Networks trained with BB-QAEs successfully denoise highly noisy entangled states, illustrating their practical utility. Furthermore, our results reveal that a quantum map trained for one type of noise exhibits cross-testing capabilities, effectively denoising states corrupted by a different noise source, underscoring the adaptability of the BB-QAE approach.

Furthermore, we conduct an in-depth analysis of the denoising mechanisms within the neural network during training by evaluating the evolution of Rényi entropy, which serves as a measure of cross-layer entanglement^38–44^. Our findings reveal that the reconfiguration of denoised entanglement predominantly occurs in the encoding part of the network, as evidenced by a layer-by-layer decrease in Rényi entropy toward the output layer. This progressive reduction in entropy is crucial to the network’s success in tolerating strong noisy flips. Consequently, these results position BB-QAEs as versatile tools for multi-qubit state preparation on NISQ devices.

Before discussing the model, it is essential to acknowledge that both QAEs and deterministic state preparation methods are fundamentally constrained by gate fidelity, a critical limitation in the NISQ era. While improvements in gate fidelity may initially appear unrelated to state denoising in QAEs^6,9,10,45,46^, they can significantly enhance the effectiveness of trained quantum maps in denoising entangled states on quantum processors. This highlights the interplay between gate performance and the robustness of denoising protocols in practical implementations.

## Quantum autoencoder training

QAE network consists of a set of interconnected qubits in layers with a bottleneck in the middle (see Fig. 1). The first (last) layer of the network represents the input (output) register. The edges connecting qubits in adjacent layers represent a quantum map from one layer to the next. There is no connection between qubits of the same layer, meaning that they may be independent on the hardware too. The network’s bottleneck is a layer with fewer qubits compared to input and output layers. From the input layer to the bottleneck, the encoder selectively retains information from the input layer to build a good encoding in the bottleneck. Initialized in the computational ground state, the decoder recovers the inputs from the state encoded in the bottleneck. Optimization of the encoder’s and decoder’s maps relies on the comparison between input and evolved states.

Our QAE is a dissipative quantum neural networks (DQNN) organized in *L* layers. Each layer *l* contains $N_l$ qubits, and each qubit in layer *l* is coupled to all qubits in layer $l+1$. Thus, we univocally denote the network’s topology as $(N_1, \cdots , N_L)$. In the middle of the symmetric structure of the QAE, we use a small sub-network instead of single-qubit layer and call it brainbox bottleneck (BB). It can be either mirror symmetric as the QAE, or asymmetric, as depicted in Fig. 1. Varying the morphology of BBs helps to understand how the bottleneck’s structure impacts outcome results on the output layer.

The quantum map on the QAE is constructed starting from the input layer and propagates the state forwards, layer by layer, towards the output layer. The unitary $U^l_j$ acts on all qubits in layer $l-1$ and *j*-th qubit in layer *l*. It changes the state of the *j*-th qubit in layer *l*. Therefore the quantum map that updates qubits in layer *l* looks like $\mathcal {U}^l \equiv \prod _{j=1}^{N_l}U^l_j$. For example consider there are $N_5$ qubits in layer 5 and $N_6$ on layer 6. The density matrix of layer 6 is initialized in the computational ground state $|0\rangle$ and its transformation depends on the state on layer 5, i.e. $\rho _{(6)}=\textrm{Tr}_{(5)} \{\mathcal {U}^{(6)} \left( \rho ^{(5)} \otimes |0\rangle \langle 0|^{\otimes N_{6}}\right) {\mathcal {U}^{(6)}}^\dag \}$. The trace isolates the state on layer *l* and dissipation equips the network with forgetfulness, a necessary condition to learning^47^. Therefore one can easily conclude that the output density matrix can be generated as follows:

1 $$\begin{aligned} \rho ^{out} = \prod _{l = 2}^{L} \mathop {\textrm{Tr}}\limits _{(l-1)} \left\{ \mathcal {U}^l \left( \rho ^{l-1} \otimes |0\rangle \langle 0|^{\otimes N_{l}}\right) {\mathcal {U}^l}^\dag \right\} \end{aligned}$$

with $\rho ^{l-1}$ denoting the partial density matrix associated to the layer $l-1$. The $N_l$ qubits in the layer *l* are initialized at the ground state $|0\rangle$.

The QAE has been trained with a (1)-BB structure to enable the reconstruction of a noise-free multiqubit entangled states^30^. The study attempts to prepare ideal GHZ-states $|\Psi _\mathrm{{in}}\rangle = (|00\cdots 0\rangle + |11\cdots 1\rangle )/ \sqrt{2}$. But noisy hardware is simulated by statistically exposing each qubit to the bit-flip noise channel $\mathcal {N}(\rho _\mathrm{{in}})$ with flip probability *p*:

2 $$\begin{aligned} \mathcal {N}(\rho _\mathrm{{in}})&= \mathcal {E}_{N_\mathrm{{in}}}(\cdots (\mathcal {E}_1(\rho _\mathrm{{in}}, p), p)\cdots ) \end{aligned}$$

with $\mathcal {E}_i(\rho _\mathrm{{in}}, p) = (1-p)\, \rho _\mathrm{{in}} \, + \, p \, X_i \rho _\mathrm{{in}} X_i$ being the bit-flip channel for the qubit *i* and $X_i$ being the flip Pauli operator. For single-qubit bottlenecks, Ref.^30^ and Ref.^37^ show that the noise tolerance of the (1)-QAE is low ($p<0.3$). We continue the analysis on BB-QAEs with larger brainbox bottlenecks.

The quantum map of the BB-QAE is divided in two parts: the encoder and the decoder. In the left wing of the network, the map $\mathcal {E}(\rho ^{in})$ of the encoder is applied on the noisy inputs and hidden layers, and compresses states in the latent space, in the brainbox^28,29,48,49^. For a brainbox bottleneck with *K* layers, we note different configurations by $(n_1, \cdots , n_K)$. For example (1,1) is a linear chain of two qubits .

In the right wing of the network, the decoder map $\mathcal {D}$ reconstructs states in their original dimension, thanks to the information encoded in the last layer of the brainbox. The output state is then $\rho ^\mathrm{{out}}_x = \mathcal {D}(\rho ^\mathrm{{latent}}_x) = \mathcal {D}(\mathcal {E}(\mathcal {N}_x(\rho _\mathrm{{GHZ}}, p))$, where $\mathcal {N}_x(\rho )$ denotes a discrete noise realization *x* of the bit-flip channel, that is a combination of flipped/not flipped on all qubits of the input layer.

The aim of the quantum map is to make the output quantum state as similar as possible to the ideal target state. In other words, a successful denoising strategy on a QAE should wash out the statistical noise encoded on the input layer from the output state. This can be measured by evaluating the fidelity of the output state $\rho ^\mathrm{{out}}$ with the ideal state $\rho _\mathrm{{GHZ}}$:

3 $$\begin{aligned} F_x(\rho ^\mathrm{{out}}_x, \rho _\mathrm{{GHZ}}) =&\langle \Psi _\mathrm{{GHZ}} | \, \rho ^\mathrm{{out}}_x | \Psi _\mathrm{{GHZ}} \rangle \nonumber \\ =&\mathop {\textrm{Tr}}\limits \left\{ \rho _\mathrm{{GHZ}}\, \rho ^\mathrm{{out}}_x\right\} . \end{aligned}$$

At each training step *n*, the average fidelity over all $N_{data}$ states $\{\mathcal {N}_x(\rho _\mathrm{{GHZ}}, p)\}_{x = 1}^{N_\mathrm{{data}}}$ defines the objective function for the network:

4 $$\begin{aligned} F(n) = \frac{1}{N_\mathrm{{data}}} \sum _{x = 1}^{N_\mathrm{{data}}} F_x\left( \rho ^\mathrm{{out}}_x(n), \rho _\mathrm{{GHZ}}\right) . \end{aligned}$$

The maximization of this function instructs the network how to perform its task. First we initialize the unitaries at random operator. Although we start input qubit states are at GHZ state and the hidden layer qubits at ground state, however considering the initial unitary maps are randomly chosen, this is equivalent to initializing the qubit states at the first round at any random state. This may not be the case during iteration as the quantum unitary map gets biased toward certain domain of parameters. The interlayer unitaries $\{U^l_j\}$ are updated layerwise and iteratively with the parameter matrix multiplication method^50,51^:

5 $$\begin{aligned} U^l_j(n + \varepsilon ) \leftarrow e^{i\varepsilon K^l_j(n)} U^l_j(n), \end{aligned}$$

where $K^l_j(n)$ is the parameter matrix derived from *F*^30^. This update rule is inspired by the gradient descent algorithms^52^ and understands gradients as the derivative of *F* with respect to each unitary. After $N_\mathrm{{it}}$ updates of the quantum map, the objective function converges to 1 if the training is successful or takes smaller positive values otherwise.

**Fig. 2:**
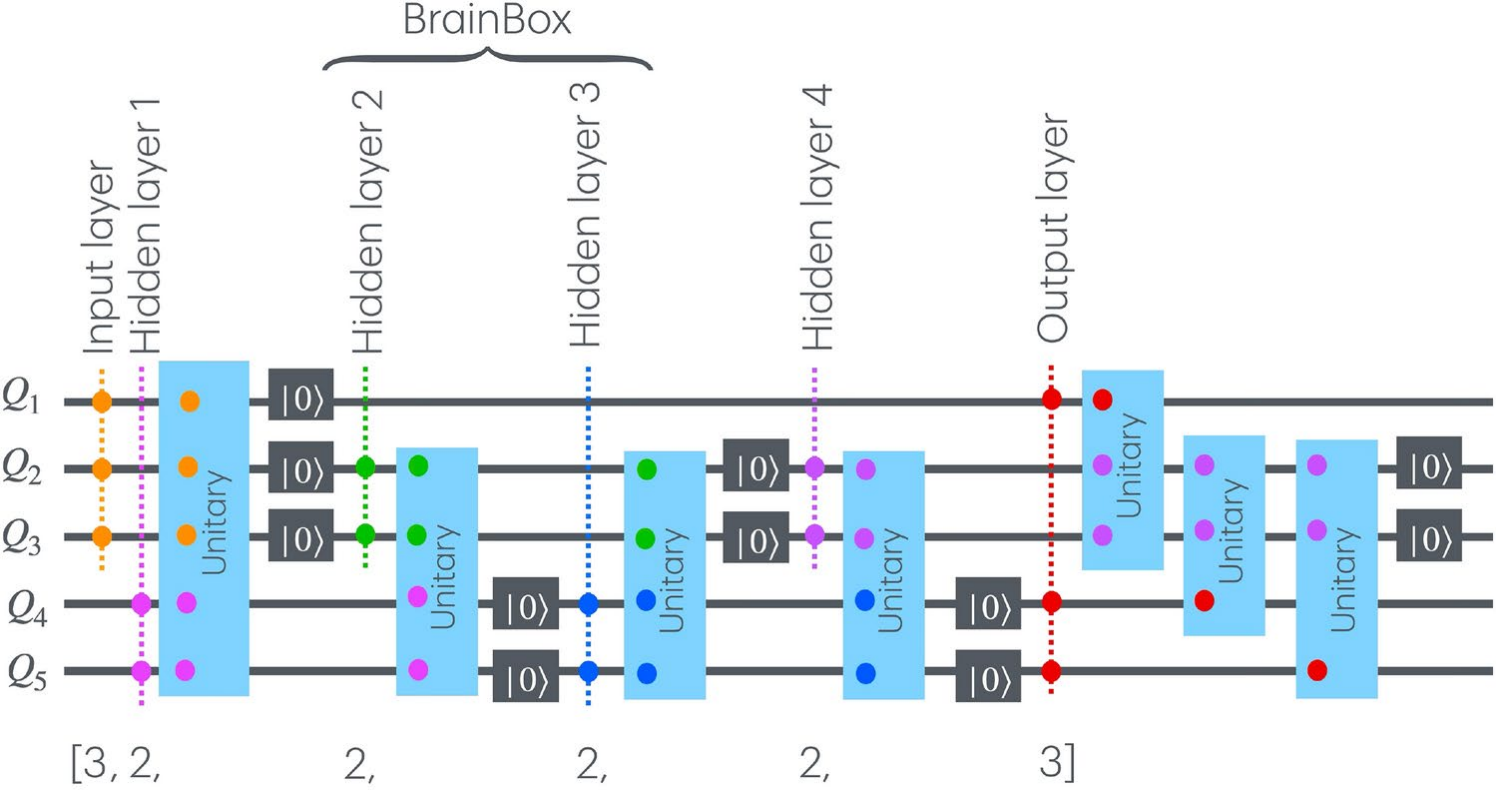
The architecture for denoising a GHZ triplet in a BB-QAE, configured as [3, 2, BB, 2, 3] with BB = [2, 2], achieves qubit efficiency through strategic resets during optimization. Each unitary interaction between layers couples marked qubits only. This method minimizes qubit use while maintaining robust denoising capability.

Denoising an *N*-qubit GHZ state might initially seem to require $2N + M$ qubits, where 2*N* accounts for the input and output qubits and *M* represents the qubits in all hidden layers. However, as shown in Ref.^37^, it is feasible to design a QAE with fewer qubits due to the fact that the cross-layer interactions take place between consecutive layers. This property allows for the resetting of qubits that become redundant after their use, enabling these qubits to be considered as part of different layers within the QAE (see Fig. 2). This approach effectively reduces the total number of required qubits to $N + \max \{h_i\}$, where $h_i$ denotes the number of qubits in the *i*-th hidden layer. For example, consider denoising a tripartite GHZ state using a BB-QAE with four hidden layers, each comprising two qubits. Nominally, this setup would require 14 qubits, corresponding to the neural network architecture [3, 2, BB, 2, 3] with BB = [2, 2]. However, since the unitary operations affect only adjacent layers, qubits can be reset and reused, reducing the lower bound of required qubits to just 5.

*Training set* Classical AEs are trained with pairs of identical input and target. Therefore in the training set we have *N* training pairs, since both input and target states need to be prepared on desired state by independent circuits. Training takes place on the input only and result is compared with the output state. Defining the desired state in quantum mechanics is practically challenging, mainly because of at least two major issue: firstly there is no access to the ideal entanglement due to their extreme noise susceptibility in electronic devices, secondly measuring fidelity requires to deal with the measurement noise. Since our aim is to denoise GHZ states, it is not possible to set them as the targets of our network, because that would imply that actual access to them is viable. Rather, we train our QAE in an unsupervised way, similarly to the training of classical AEs. Therefore classical AE training does not apply in QAEs, as we do not have access to the specific noise affecting a state. Therefore, we train our QAE on (*x*, *y*) pairs, where *x* and *y* are drawn from the same noisy distribution.

We define a virtual training set on a classical computer, enabling the simulation of the optimization process before deployment. Once the training is completed, the resulting optimal quantum map can be implemented directly on a quantum processor in a single operation. This follows the training methodology outlined in Ref.^30^. During the training phase, input and target states are drawn from the same predefined training set. A key observation is that if the majority of states within the training set are identical, the optimization process naturally biases the quantum map towards reproducing the dominant state, i.e. this reduces the cost function $1-\bar{F}$ with the average fidelity defined Eq. (4). This occurs because the cost function is minimized by aligning the output state with the majority representation in the training set. Consequently, the quantum map converges to one that maximally preserves the characteristics of the predominant state in the training set, reflecting an inherent compatibility with its statistical distribution.

**Fig. 3:**
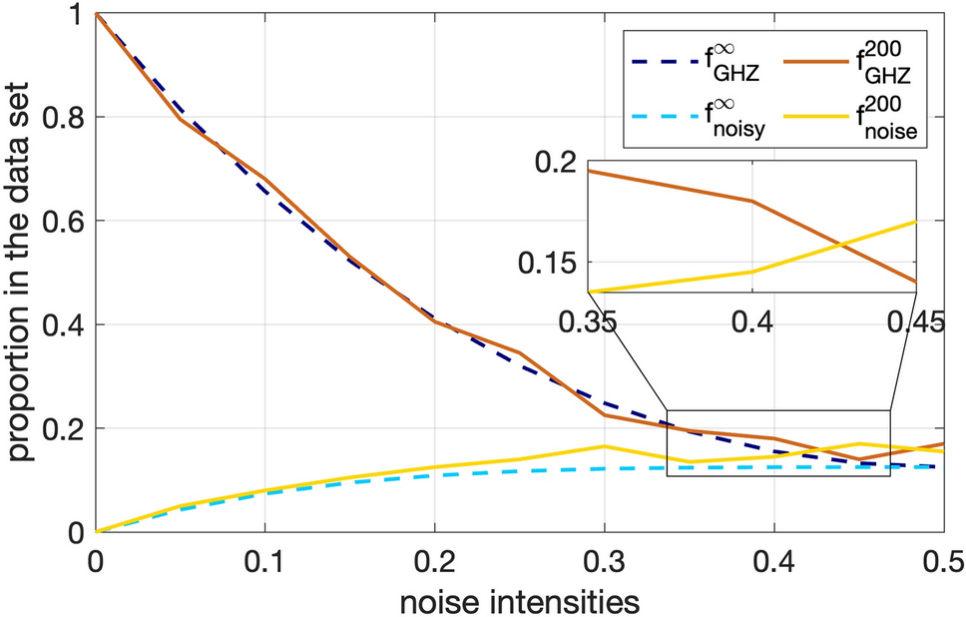
Training data set: The distribution of 4-qubit GHZ and states other than GHZ in the training set of infinite size in dashed, versus 200 samples size in solid lines. In the infinite size case, the distribution of GHZ for all noise probabilities *p* dominates, while in the finite set at large *p* sometimes artefacts prevents the dominancy of GHZ over other states due to strong noise channels.

As the size of the training set becomes finite, deviations from the ideal distribution alter the training. Dashed and solid lines in Fig. 3 compare distributions for ideal states and the next most probable noisy state for training sets with infinitely many and 200 states respectively. In limited data set, the ideal GHZ state occurs less often in the finite data set than the next most probable noisy state in the vicinity of $p\sim 0.4$. Thus for such data set, the QAE training can only help to boost tolerance threshold up to where GHZ state constitute a majority of the training data. While the training data ultimately imposes an upper bound on the tolerance that can possibly be achieved, the (1)-QAE performs sub-optimally and its tolerance does not depend on the training data. For the multi-qubit BB, he scaling of the generalization error with the size of the training data set is consistent with the results in Ref.^53^.

Before discussing results, let us briefly outline the characteristics of QAE-based state preparation and clarify its goal as compared to the parameterized state preparation. Firstly a training set of noisy states with single-qubit noise probabiloity *p* is generated. Secondly, a state from the set, say *x*, is chosen on the input later and the QAE training takes place upon applying a set of random cross-layer unitary gates between consecutive over a network similar to Fig. 2, and eventually the fidelity of the output state is detemined by comparing its distance from another randomly selected state from the set *y*. Thirdly, the quantum map backpropagates to be optimized in a way that at every iteration the fidelity increases. This process takes place on a classical computer, but the result of optimized state can be used on a quantum computer once. This makes the QAE denoising process different from the parametrized state preparation; the former is a noise-learning process, while the latter is a deterministic process and can be used without revealing any information about noise.

## Results

In our model, every qubit of the state is flipped with a certain probability *p*. Denoising a four-qubit GHZ state has been previously performed in Refs.^30,37^ on some symmetric input/output QAE network examples, such as a simple single-qubit bottleneck with additional hidden layers (4,2,1,2,4) and without them (4,1,4), and (4,1,4,1,4) network which is twice (4,1,4). Each training employs 200 GHZ states exposed to noise of certain bit-flip probability *p*.

For each *p* the performance assessment is evaluated by comparing mean fidelity function before and after applying denoising quantum map. These QAEs can ideally denoise GHZ state up to the tolerance noise $p^*=0.3$, see Fig. 2 in Ref.^30^. We use the same scheme on different network topologies, and our aim is to understand under what topology or connectivity conditions noise tolerance $p^*$ can be improved beyond the weak limit of 0.3. This enable QAEs to denoise entangled states under harsh noise exposure. In addition, it gives better prospects to scale up the inputs.

### Tolerance threshold

**Fig. 4:**
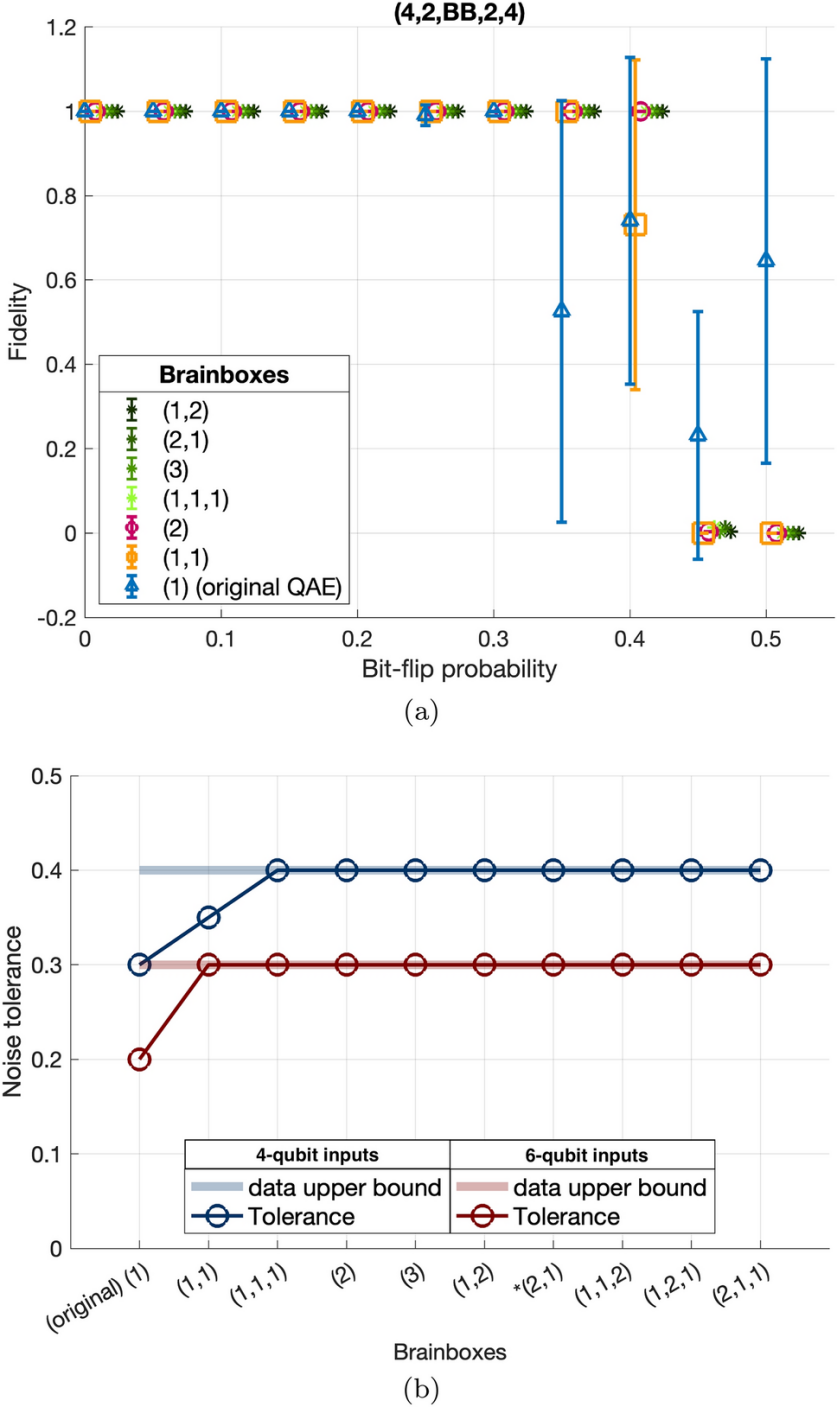
(**a**) Testing fidelity: Average output state fidelity over a range of noisy test states with noise probability *p*. The error bars indicates the absolute value of standard deviation in the data about average fidelity. When it is large, it indicates that some noise realizations do not reach high fidelity states after denoising while some do. (**b**) Tolerance thresholds: The noise probability that returns output states with at least 99% fidelity with the ideal GHZ state. Various networks with 4- and 6-qubit input/output layers and different BBs have been tested. Some brainbox bottleneckes make large improvements in the network tolerance threshold.

Our QAEs contains two symmetric hidden layers each with 2 qubits next to the input and the output, so that the topology is (4,2,BB,2,4) and (6,2,BB,2,6). We consider the brainbox bottlenecks listed in Fig. 4. The BB examples are the following symmetric sub-networks (3), (2), (1), (1,1) (1,1,1) and the asymmetric BB sub-networks (1,2) and (2,1). In order to make a clear comparison between how fast each brainbox makes its way to an optimized quantum map, we start all these networks at the same initial map and we update the map 200 times. Among these examples, the (1)-QAE represents the original QAE from Ref.^30^.

After training the above-mentioned BB-QAEs with training sets of 200 noisy GHZ states and different bit-flip probabilities *p*, testing checks whether the network denoises states it was not trained with. The optimized quantum map is applied to some new noisy GHZ-states. Though the noise realizations differ, the noise channel is the same as during the training. The output states are compared with an ideal GHZ state using the fidelity function 4.

The result is shown in Fig. 4a. We define the **tolerance threshold** to be the largest probability the network can recover from and show the fidelity nearly 1 at the end of training. One can see that the simple brainbox bottleneck (1) used in Ref.^30^ can tolerate only noise probability up to 0.3, while the (1,1)-QAE can increase the threshold slightly to 0.35. The other brainbox bottlenecks push the tolerance threshold up to 0.4 and perform equally well with respect to fidelity.

A low tolerance threshold QAE can make issues in two ways. To make the QAE useful on the current NISQ devices, it must operate well at intermediate noise scales, close to the tolerance thresholds. In addition, errors are more likely to occur on larger states. Therefore, noise resilience must be improved. For this aim, we compared the results of (4,2,BB,2,4) and (6,2,BB,2,6) networks. We plot the respective thresholds in Fig. 4b. By increasing the number of input qubits from 4 to 6 while aiming at GHZ-states, the noise tolerance on a simple single-qubit bottleneck shows a large drop off by 0.1 from $p^*=0.3$ to 0.2. This raises concerns about the scalability of denoising: by adding more qubits to the inputs, the noise tolerance shrinks, in other words the QAE becomes more fragile and unable to recover the ideal target state.

For this study, a size-200 training set already shows disparities in improvements in tolerance due to the network topology. Fig.4b shows that in a network of 4-qubit inputs all brainbox bottlenecks, except (1) and (1,1), equivalently perform with higher tolerance. Adding two qubits to the input results in the reduction of noise tolerance by 0.1 unit, however in this case still all BBs except the single-qubit bottleneck (1) reveal higher tolerance. Another important lesson from the study is that qubit configurations in BBs contribute to the tolerance. For example in the case of 4-qubit input, a brainbox with two qubits in separate layers (1,1) yields a sub-optimal tolerance at 0.35, while stacking them in a single layer (2) saturates the data limit at 0.4.

### Training impedance *Z*

In section 3.1, we found that most of multi-qubit brainboxes we used in the (4,2,BB,2,4) and (6,2,BB,2,6) networks maximize the bit-flip noise tolerance $p^*$. Differences highly depends on the BB topologies, some of which makes the training less costly.

During the optimization of the training map, at some step onward the fidelity of the output layer starts to grow monotonically. Let *n*(*F*) denotes the step at which the training achieves the fidelity equal or above *F* in the output layer. Consider that each BB-QAE is trained for $N_\mathrm{{it}}$ iterations. We start the training at any initial state from the training set and this makes some slight changes in the step number at which the fidelity *F* is reached. Initializing at all states of taining set and taking the average number of the step at which the fidelity *F* is reached determines $\bar{n}(F)$. For a fixed value of iteration number $N_\mathrm{{it}}$ for all trainings, we define the he **training impedance** as the normalized $Z(F) = \bar{n}(F) / N_\mathrm{{it}}$. The smaller training impedance indicates less resistivity against the training in the network, i.e. faster training. For a fixed $N_\mathrm{{it}}$ this quantity can serve as a measure to identify the faster trainability among BB-QAEs.

**Fig. 5:**
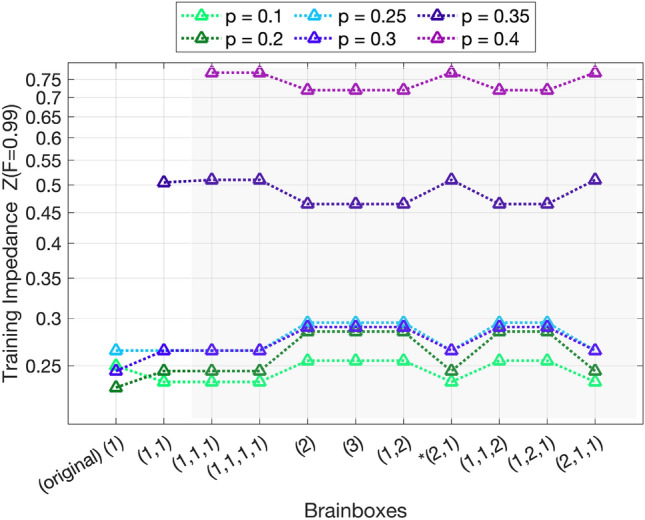
Training impedance for the optimization of (4,2,BB,2,4) networks. As the noise intensities grow, the optimization is more demanding. Some BBs show to be less efficient to rapidly gain high fidelity in the output state. These results are robust and remain unchanged for any randomly selected initial mapping, as described before Eq. (5)..

Figure 5 shows $Z(F=0.99)$ in several networks of different BB topologies on the x, each BB-QAE trained for different noise probabilities *p*. The result indicates that training impedance depends on the fidelity limit, training noise probability, brainbox, and input qubits, i.e. $Z=Z(F, p, \textrm{BB}, N_\mathrm{{in}})$.

**Fig. 6:**
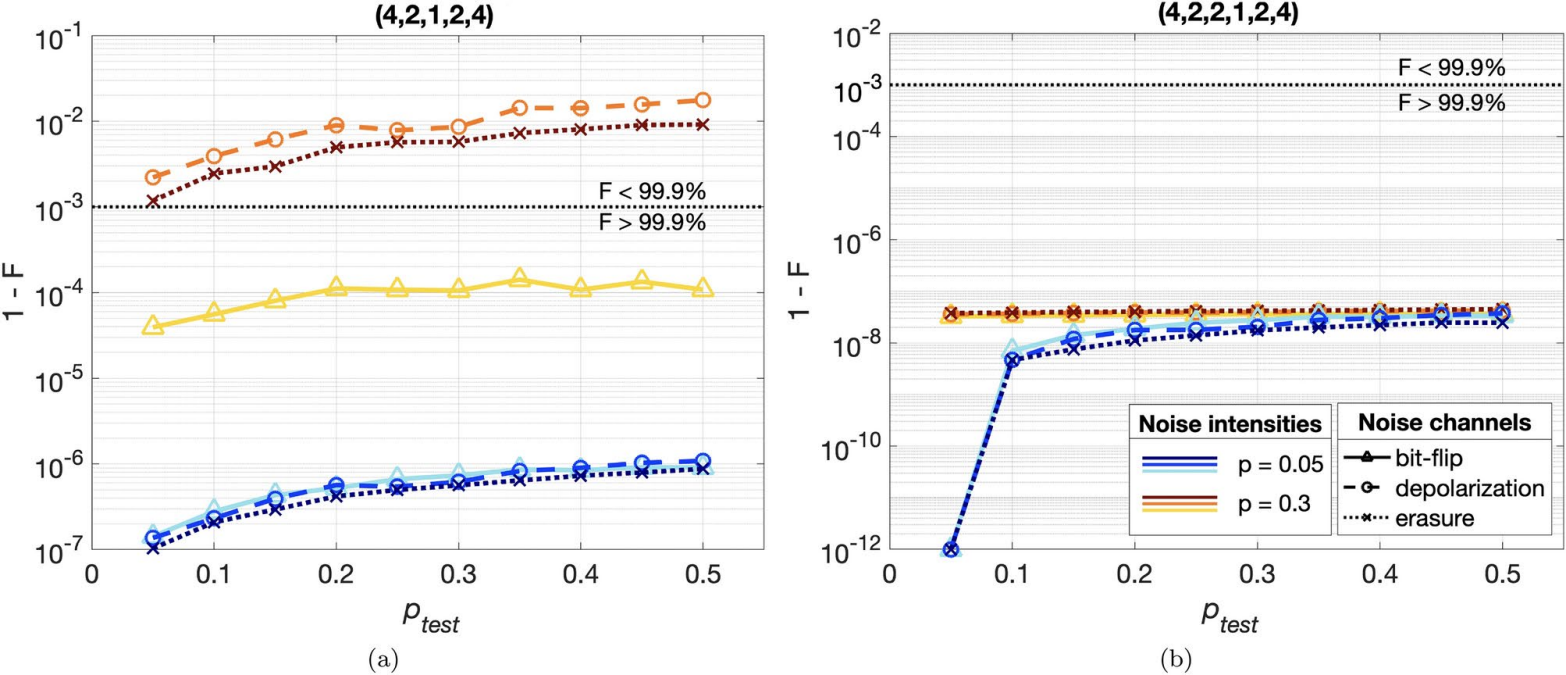
Cross-tests results for two networks: (4,2,1,2,4) and (4,2,2,1,2,4) associated to (1) and (2,1) brainbox subnetworks. Three noise channels were implemented with noise intensities $p_{test}$: the bit-flip channel (full lines), the depolarizing channel (dashed lines) and the erasure channel (dotted lines). The (1)-QAE shows more sensitivity noise in the test states: for the same training probability $p_{train}$, the reconstruction error fluctuates and larger errors occur on unfamiliar noise channels. In contrast, the map optimized by the (2,1)-QAE treats all noise channels and intensities equally. The outputs of the BB-QAE lose dependency on the noise it was trained with. In addition, the reconstruction error over the weak noise regime is lower compared to the (1)-QAE.

Results for (4,2,BB,2,4) networks are summarized in Fig. 5. For noise probabilities $p \le 0.3$ the impedance factor *Z*(0.99) in all BB networks remains relatively small nearly between 0.23 to 0.30; meaning that all networks at these noise probabilities can easily find their way to fidelity above 0.99 within the first third of the training. Some BBs such as (2), (3), (1,2), (1,1,2), (1,2,1) are slightly slower in gaining high fidelity. However the very same network under harder noise of $p\ge 0.35$ have an advantage during the training and optimization is almost 5% faster than in other networks.

The selection of a suitable brainbox is based on the trade-off between the gain in fidelity and the loss in computational speed. At low noise intensities such as $p=0.1$, linear brainboxes (1,1),(1,1,1) and (2,1) accelerate the training compared to the single qubit box (1). Longer brainboxes also protect the network against overfitting (see section 3.3). Between p=0.2 and 0.3, multi-qubit brainboxes cause a small computational overhead that is minimized by the linear architectures. Above the (1)-QAE’s tolerance threshold, wide brainbox structures such as (2), (3), and (1,2) improve the training efficiency compared to the linear ones. Thanks to a larger amount of parameters, they efficiently capture subtle patterns in the training states, as in the over-parametrized regime^54,55^.

Similar graphs for (6,2,BB,2,6) networks are shown in the Supplementary Material.

### Cross-testing

In previous sections, the testing data set was generated under the same noise channel as during the training of the quantum map. A generalization of this approach has been described in Ref.^37^, in which the QAE is trained using a noise channel with parameter *p* and is tested with the same channel with different parameter $p'$. In this section, we evaluate the BB-QAEs with a generalized cross-test: the testing data originates either from the same noise channel with different intensity, or from a different noise channel.

We consider two BB-QAEs with brainboxes (1) and (2,1). Though these two brainboxes have similar impedance factors (see Fig.5), they differ by their tolerance threshold (Fig.5). We train them both with bit-flip noise at intensities $p_\mathrm{{train}}=0.05$ and 0.3. After the training is completed, we use the final map to test noisy input GHZ states generated by one of the following three noise channels with independent noise intensities $p_\mathrm{{test}}$: (1) bit-flip channel defined in Eq. (2), (2) depolarizing channel $\mathcal {E}_i^{dep}(\rho , p_\mathrm{{test}}) = (1-3p_\mathrm{{test}}/4) \rho + p_\mathrm{{test}}/4 (X_i \rho X_i + Y_i \rho Y_i + Z_i \rho Z_i)$, which can add a relative phase between $|00\cdots 0 \rangle$ and $|11\cdots 1\rangle$ of GHZ-states and can rotate each qubit around an arbitrary axis, and (3) erasure channel that by probability $p_\mathrm{{test}}$ replaces the state of a single qubit in the GHZ state with a random state $\alpha |0\rangle + \beta |1\rangle$, otherwise it remains unchanged,^56,57^. In the latter, since all $\alpha$’s and $\beta$’s are different for each noise realization, the map is challenged to reconstruct GHZ-states starting from any possible pure quantum state. In Fig.6, we evaluate the generalization error with the reconstruction error $R(\{\rho _x^\mathrm{{out}}\}, \rho _\mathrm{{GHZ}}) = 1 - 1/N_\mathrm{{test}}\sum _{x = 1}^{N_\mathrm{{test}}} F_x (\rho _x^\mathrm{{out}}, \rho _\mathrm{{GHZ}})$ where $N_{test} = 200$ is the number of states in the testing data set.

For both network morphologies, training with weak noise yields almost perfect generalization to all three noise channels over a large range of probabilities. In Fig. 6a,b and Fig. (??), reconstruction error is kept in the negligible range.

We repeat the same cross-testing procedure at the tolerance threshold of the (1)-QAE. In Fig. 6a, this network recovers from the bit-flip channel with reconstruction error close to 0.001. In contrast, states affected by the erasure and depolarizing channels cannot land on ideal GHZ state with high fidelity ( higher than 99.9%). This is a sign of overfitting, since the discrete states in the former case are already represented in the training data set. The two remaining noise channels add states that are new to the network. In this respect, the noise tolerance measure in Fig. 4 is deceitful to the extend that the last optimized map works solely on the training states.

Training the (2,1)-QAE with $p_\mathrm{{train}}=0.3$, ie below its tolerance threshold, enables the full recovery of erroneous states irrespective of the noise channel tested, at all $p_\mathrm{{test}}$. This is possible due to the fact that the extended network has access to the dominating fraction of ideal GHZ states, which brings advantages in the cross-tests as well. One can think of the BB structure as a magnifying glass that makes it possible to distinguish targets from noise even when they are close to one another, by creating a better encoding of inputs in its last layer.

### Rényi entropy flow

A key property to measure in engineered quantum systems is entanglement: in contrast to their classical counterparts, quantum algorithms can generate large amounts of entanglement between parts of the system^58,59^. Entanglement during the learning phase in a QAE changes internally across layers. It allows delocalizes information in the network and steers the training towards the optimal condition of having a separable output. In order to observe its contribution to the training, some measures of entanglement have been tested, such as entanglement witnesses^60^ and von Neumann entropy^61^. Similar to any many-body quantum system, measuring the entropy of different partitions provides a way to probe its entanglement structure.

Here, we evaluate the second-order Rényi entropy since it can capture long-range entanglement^38–41^ as well as dissipation mechanisms^42–44^. Rényi entropy can serve as a measure for probing and characterizing brainbox bottlenecks. A slow entropy growth in a layer or in a part of the network can be used to identify localization in a subset of the network^62^.

**Fig. 7:**
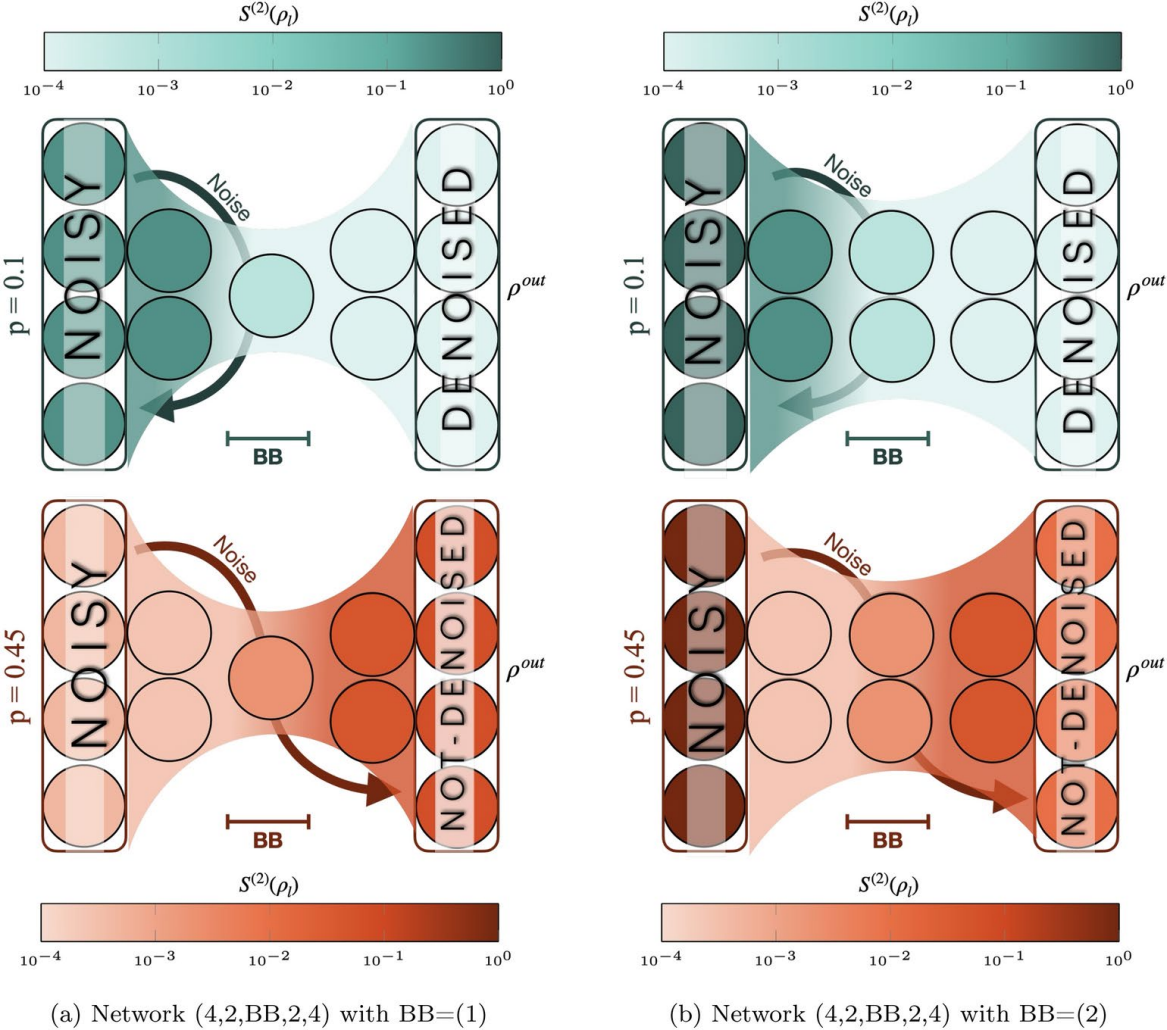
Layerwise Rényi entropy . Darker colors indicates larger entropy of noisy mixed states. We study a single-qubit brainbox BB=(1) in (**a**), and a double-qubit layer brainbox BB=(2) in (**b**). IN the first rwo on both (**a**) and (**b**) single qubit flip probability is $p=0.1$ and this makes entropy decrease from let to right in the network. In this case one can see noise is localized in the encoder and is blocked away from the brainbox; i.e. this is how both brainboxes can filter out noise at the bottleneck. In the lower rown of (**a**,**b**) we consider stronger flip probability $p=0.45$ than the tolerance of all BB-QAEs, and we see input noise leaks out of the bottleneck to the right side and noise accumulates in the decoder and output layer. For all these evaluations we first initialize the input layer at the training set, then apply the optimum unitary map we trained to denoises the output layer state, and evaluate the density matrix, and finally by tracing out some layers we evaluate entropy of layers. We take average of the entropy by evaluating it at different initial state.

For a bipartite system $\mathcal {S}$ with subsystems A and B and total density matrix $\rho$, second order Rényi entropy is $S^{(2)}(\rho )=- \log {(\mathop {\textrm{Tr}}\limits \{\rho ^2\})}$. When equal to zero, it indicates that $\mathcal {S}$ is pure and independent from any environment. Typically, entropy of the whole BB-QAE is zero at all iterations because the system is isolated from the environment and therefore in a pure state. Moreover, second order Rényi entropy can be evaluated for any subsystem in $\mathcal {S}$, eg. A, based on the associated partial density matrix $\rho _A = \mathop {\textrm{Tr}}\limits _B (\rho )$: $S_A^{(2)}(\rho _A) = - \log {(\mathop {\textrm{Tr}}\limits _A\{(\rho _A)^2\})}$. Consequently, at each training step, in a BB-QAE with *L* layers, the entropy of layer *l* reflects the presence of entanglement between the layer *l* and the remaining $L-1$ layers in the network. Since the result may depends on initial state, we initialize the input layer at any of the states in training set, then apply the optimum unitary map and using the output density matrix we evaluate the average value of entropy. Therefore the average second order Rényi entropy of the layer *l* for a training set of size *N* is defined as

6 $$\begin{aligned} S_l^{(2)} = \sum _\mathrm{{training\ set}}- \log {(\mathop {\textrm{Tr}}\limits _l\{(\rho _l)^2\})}/N, \end{aligned}$$

with the partial density matrix of layer *l* being $\rho _l = \mathop {\textrm{Tr}}\limits _{k \ne l}\{\rho \}$ for $k=1,\cdots L$ and $\rho$ is the state of the whole BB-QAE.

In particular, at each iteration, the entropy of layer *l* can be evaluated using Eq.(6) after applying the respective unitary $\mathcal {U}^l$. During the training, we compare the evolution of layer-wise entropy in a (1)-QAE for both weak ($p=0.1$) and strong ($p=0.4$) noise in the input GHZ states (see Fig. 4a,b in Supplementary Material). During the learning phase, entropy is redistributed within the network. In the first steps, it undergoes steep growth, especially in the last layer. In the subsequent iterations, entanglement vanishes exponentially in the decoder’s layers, while it is only slightly suppressed in the encoder, resulting in entropy inversion.

Entropy after optimization is compared for BB-QAEs with BB=(1) and BB=(2) below and above the tolerance threshold, at $p=0.1$ and $p=0.45$ respectively (Fig. 7). In a BB-QAE with bit-flipped GHZ state on the initial layer, successful denoising not only raises fidelity of the output states, but also improves its separability. Therefore, training inverts entropy in the network and shifts noise from the decoder to the encoder. The bottleneck seals it away from the output layer.

In contrast, failure to denoise the inputs can take two forms. In Fig. 7a, instead of concentrating noise in the encoder, the training yields high entanglement in the last two layers, while the encoder remains almost independent. As in Fig.7b, the inversion of entropy can be favorized by using larger BB structures. In this case, the training improves noise concentration, but the bottleneck seal seems too porous to lock noise out of the decoder, resulting in poor denoising.

## Conclusion

We have presented an in-depth study of various brainbox structures for the bottleneck in a quantum autoencoder used to denoise entangled quantum states. Training a QAE single-qubit bottleneck has been studied in Ref.^30^. This bottleneck can come with only limited tolerance against bit-flip, depolarizing, and random unitary noise channels. Scaling the inputs size from 4 to 6 qubits makes the training more greedy in data, and deteriorates the denoising performance rapidly.

We identified two mechanisms behind the limitation of noise tolerance. (1) The finite size of the training data set causes statistical deviations from the ideal noisy state distribution expected from the bit-flip channel. It imposes an upper bound on the maximum tolerance the BB-QAE can achieve. This upper bound depends on each training data set. (2) The study of Rényi entropy shows that the single-qubit bottleneck is unable to seal noise away from the output state, and therefore to carry out its denoising task.

We compared the simple QAE with multi-qubit brainbox bottlenecks, most of which brought significant elevation of tolerance. When qubits are added to the input and output layers, the relative improvements are maintained. If a brainbox bottleneck can endure stronger noise compared to another brainbox, adding more qubits to input state maintains the superiority of the former one.

Some bottlenecks show similar tolerance threshold against noise. This raises an important question: What other features can make a brainbox more suitable than the other ones? To address this question, we compare training impedance between brainboxes. For this purpose, we evaluate the training impedance *Z*(0.99), which indicates what minimum percentage of the training process is required to achieve a fidelity above 99% in the output. The result has been summarized in Fig. (5) and show that the training impedance depends not only on the bottleneck, but also on the training noise probability *p*. Below bit-flip probabilities $p=0.3$, linear brainboxes such as (1,1) are favorable to a more efficient training. In contrast, between $p=0.3$ and $p=0.4$, non-linear brainboxes such as (2) or (2,1) are most economical to train.

We evaluate the Rényi entropy of network layers at each optimization step to show how nonlocal entanglement between layers evolves and impacts the outputs fidelity. Results show that in networks below their tolerance threshold, entropy becomes localized in the encoder of the BB-QAE, so that much less noise passes through the bottleneck to the decoder. This usually leads to outputs states that have high fidelity with the target and that are separable from the network. Some examples were given in Fig.7: in successful training, noise is blocked off from the bottleneck, while in unsuccessful training noise penetrates through the bottleneck. The absence of separability of the output indicates the presence of layer-to-layer stray coupling between hidden and output layers, which eventually does not allow its fidelity to rise higher.

In connection to NISQ devices, QAEs are resilient to input layer noise and therefore they provide the potential to generate ideal entanglement on noisy gates and qubits. A QAE with complex bottleneck and more qubits and parameters in general seem advantageous for denoising, because such a complex structure provides the possibility to separate encoder and decoder. However detailed analysis shows that less resourceful brainboxes can be found with the same performance as a complex one. Testing the network with the depolarizing and erasure channel proves that some bottlenecks can keep their superiority over the whole trainable range. We expect that these differences will remain when selecting different quantum target states.

There are many avenues worth exploring with this QAE in the future. In particular, since the network can function as a quantum error correcting code (QECC), it would be interesting to benchmark it again other state-of-the-art QECCs, notably to compare its denoising accuracy as well as the time and resources involved. In this paper, we were limited by computational resources, so we could only fully test [2,1,2] and [3,1,3] networks. In the future, it would be use- ful to explore the effectiveness of the network with different topologies.

One of the main obstacles against implementing QAEs in scaled up input states is the required high connectivity in the network that is inaccessible on the current processors. An alternative is to train a map with missing connections^30^.

## Supplementary Information

Supplementary Information.

## Data Availability

The codes generated and/or analyzed during the current study are not publicly available due to limitations from funding resources but are available from the corresponding author on reasonable request.
