# Supplementary material for: Error mitigation in brainbox quantum autoencoders
Source: Sci Rep. 2025 Jan 17;15:2257. doi: 10.1038/s41598-024-84171-z (PMC11748736; doi:10.1038/s41598-024-84171-z)
Supplement: Supplementary file 1 — Supplementary Information. [file 41598_2024_84171_MOESM1_ESM.pdf]

Supplementary Materials: Error mitigation of entangled states using brainbox quantum autoencoders

Joséphine Pazem[1,2] and Mohammad H. Ansari[1]
[1] *Peter Grünberg Institute, Forschungszentrum Jülich, Jülich 52428, Germany*
[2] *Institute for Quantum Information, RWTH Aachen University, D-52056 Aachen, Germany*

Appendix A: Denoising a 6-qubit input GHZ states on (6,2,BB,2,6) networks

In this section we present results related to denoising 6-qubit input GHZ states.

a. *Training procedure*

The input layer is initialized with 6-qubit inputs, while the rest of the network is initialized in the ground state. The network is trained with a set of 200 noisy GHZ states and bit-flip probabilities p. After the training is over, the optimized quantum map is used to test the performance on some new noisy GHZ states the network was not trained with. The result is repeated for 200 test states at different p values between 0 and 0.5. For every choice of brainbox, we evaluate the output state fidelity. Results are plotted in Fig.1.

As discussed in the main text, in the limit of infinite size data set, the distribution of GHZ and non-GHZ states is such that the amount of GHZ states is always larger than that of non-GHZ except at $p = 0.5$. This distribution in Fig.1(b) is shown in dashed line. Reducing the size of our training data to a finite value makes zigzag deviations about the ideal distribution. Around $p = 0.35$, the finiteness disorder reverts the superiority of GHZ state which deceives the network into recovering an undesired target state. Solid lines in Fig.1(b) shows the training data we used to denoise a 6-qubit QAE.

b. *Training impedance*

Results for (6,2,BB,2,6) networks listed in Fig.1(a) show the tolerance threshold for denoising outputs. However, some complex BBs such as (1,2), (3) reach the same tolerance than simpler BBs, such as (1,1), (2). In order to understand which BB is more efficient, we evaluate training impedance in the networks. Results can be found in Fig. (2). For all p values within the range indicated in the plots, the network (1,1) and (1,1,1) have less resistivity against training compared to the network (2) and (3), and even (1,2).

c. *Cross testing*

In the section the result of cross testing of the (6,2,1,2,6) network with brainbox (1) is discussed. As mentioned in the previous appendix sections, the (1)-QAE network can tolerate noise in the domain of $p \leq 0.2$. In this range of noise strengths, as shown in Fig. (3) the network carries an intermediate impedance to training, which makes it efficient for training. Training the network with bit-flip noise channel of $p_{\text{train}} = 0.05$ trains the network based on a dominant subset of GHZ states in the training data set.

This training makes the network resilient to (1) bit-flip, (2) depolarizing, (3) erasure channels in a large domain of noise strength $p_{\text{test}} < 0.5$. However training the network with input noise probability beyond the network tolerance makes the network confused about the identity of the dominant subset in the training data set. This suppresses the fidelity of output state to $< 90\%$.

Appendix B: Entropy evolution

In this appendix we list some result on the time evolution of entropy during training steps. We consider the network (4,2,1,2,4) with (1)- bottleneck. On this network we start the input layer (layer 1) with a set of noisy GHZ states with noise strength p. All other qubits in other layers are in the ground state. We initialize a quantum map at random

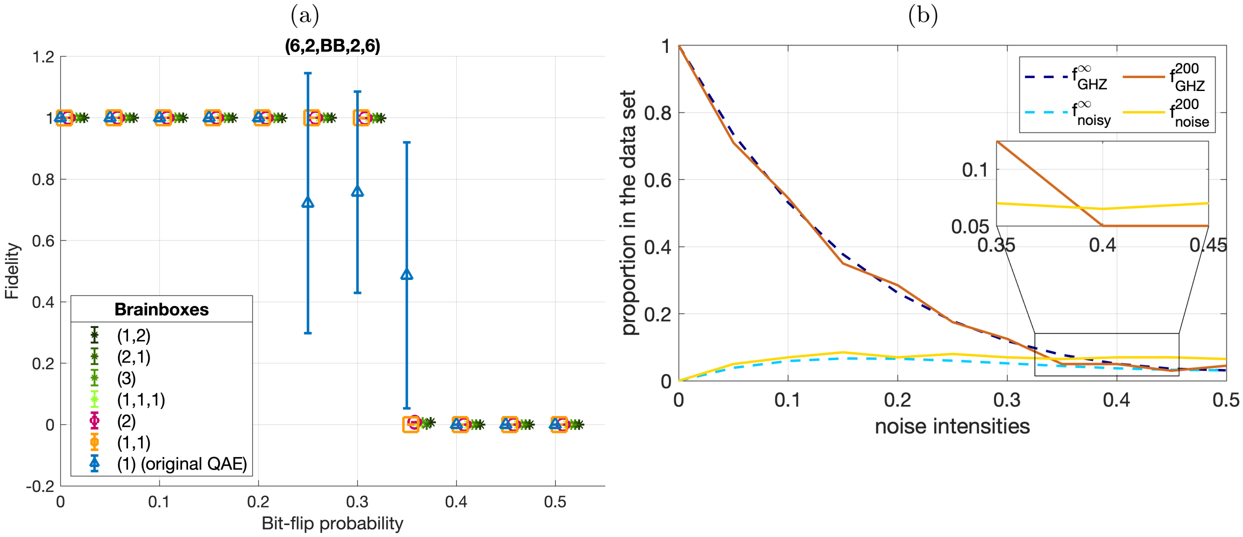

FIG. 1: (a) Testing fidelty: Average output state fidelity over a range of noisy input test states with noise probability p. The error bars indicates the absolute value of standard deviation in the data about average fidelity. (b) Training data set: The distribution of 6-qubit GHZ and non-GHZ states in infinitely many samples (dashed line) versus finite 200 samples (solid lines). In the infinite sample case the distribution of GHZ for all noise probabilities p dominates, while artefacts in the finite data set prevents the dominance at strongly noisy channels.

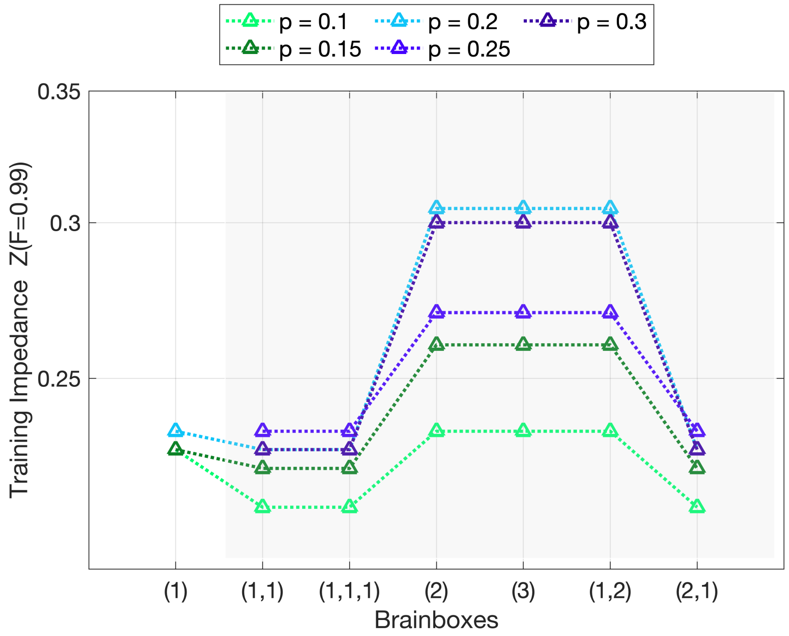

FIG. 2: Training impedance for the optimization of (6,2,BB,2,6) networks. In contrast to the 4-qubit inputs, linear BBs perform best at all noise intensities, except for the (2,1)-configuration.

and optimize it iteratively to create ideal GHZ states on the output layer (layer 5). At each step we evaluate total network density matrix and by tracing out the irrelevant layers, we evaluate the second order Rényi entropy for each layer. The result for $p = 0.1$ can be seen in Fig. (4 a) and for $p = 0.45$ in Fig. (4 b).

As expected, all layers start from zero entropy and quickly raise their entropy as they capture mixed state from the noisy input. Even as one can see the output layer (layer 5) shows a large entropy after a few steps of optimization.

Continuing optimization lowers the entropy of the decoder (layers 4 and 5) much faster than in the encoder (layers 1 and 2). The end of the denoising processes have been magnified in the two insets in (a) and (b). One can see that in the weak noise regime of (a) with $p = 0.1$ layer 5 carries zero entropy, which makes it a separable state from other

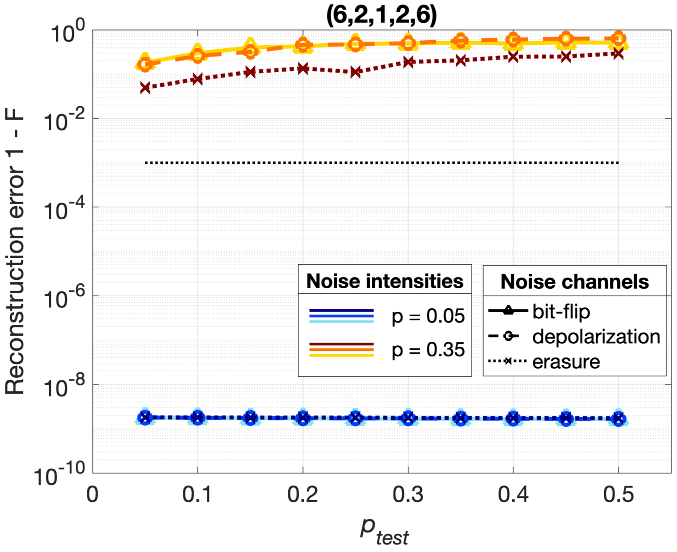

FIG. 3: Cross-tests results for two networks: (6,2,1,2,6) associated to the brainbox subnetwork (1). Three noise channels were implemented with noise intensities p_{test}: the bit-flip channel (full lines), the depolarizing channel (dashed lines) and the erasure channel (dotted lines).

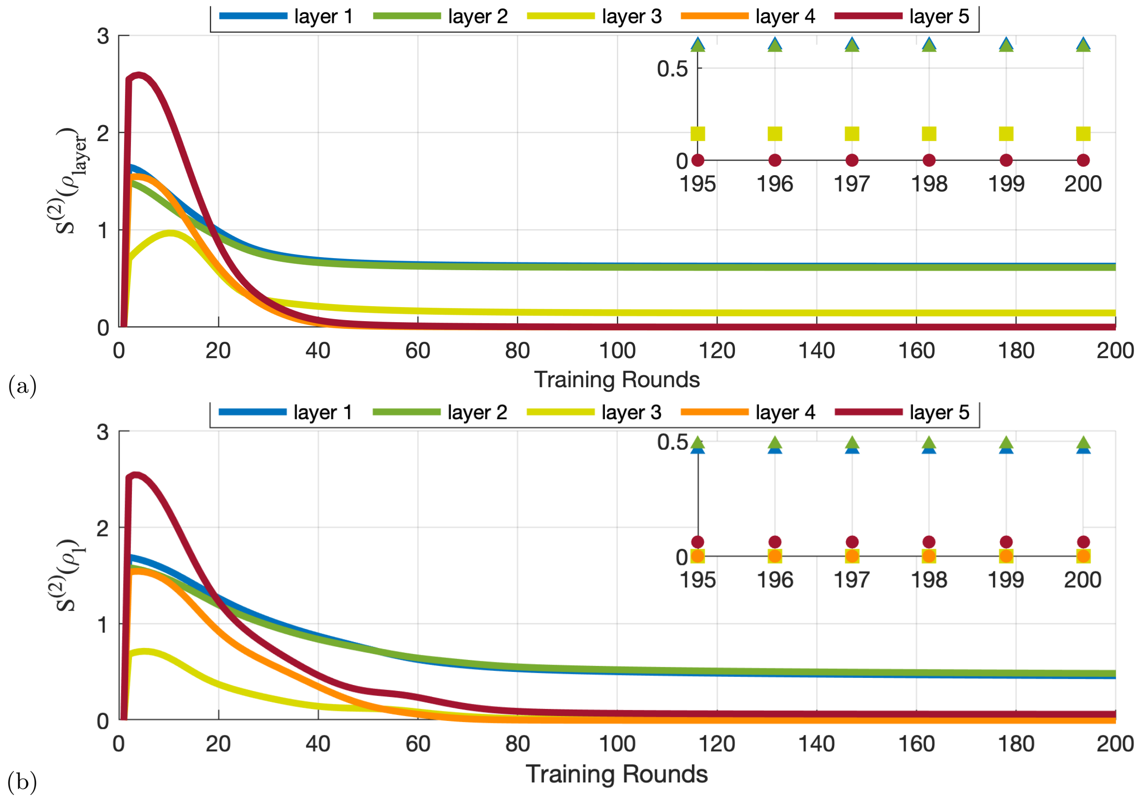

FIG. 4: Rényi entropy flow in individual layers of (4,2,1,2,4) network during its evolution during the training (x-axis). The bit-flip probability p of the noisy input GHZ states is 0.1 in (a) and 0.4 in (b).

layers. In the strong noise regime (b) with $p = 0.45$, entropy of the output layer is finite and larger than that of the bottleneck (layer 3), which makes the state entangled to other layers and therefore being affected by input noise. This prevents this network to land on stable high fidelity GHZ state due to stray couplings.
